# Albemarle–Pamlico Sounds revealed and stated preference data

**DOI:** 10.1016/j.dib.2015.01.006

**Published:** 2015-02-13

**Authors:** John C. Whitehead

**Affiliations:** Department of Economics, Appalachian State University, Boone, NC, United States

## Abstract

In this article we describe the contingent valuation and behavior methods scenario developed in the 1995 Albemarle–Pamlico Sounds Survey. The survey elicits revealed and stated preference recreation behavior data which are used to estimate the value of water quality improvements [4,8]. The survey elicits willingness to pay data which are used to conduct a split-sample scope test [7]. The data are used to jointly estimate revealed and stated preference recreation and willingness to pay data [2,6]. The data has been, and can continue to be, used to investigate econometric specification [3], bid design and other nonmarket valuation issues. The data have been used as illustrations and examples in three books that develop nonmarket valuation methods [1,5,9]. Data are supplied with this article.

**Specifications table**

**Table:** 

Subject area	*Economics*
More specific subject area	*Nonmarket valuation*
Type of data	*Excel file*
How data was acquired	*Telephone survey*
Data format	*Raw*
Experimental factors	*None*
Experimental features	*The data contain a split-sample scope test*
Data source location	*The eastern region of North Carolina*
Data accessibility	*Data is with this article*

**Value of the data**

• The data contains contingent valuation method data with a split-sample (external) scope test.
• The data contains contingent behavior data that allows a test for hypothetical bias.
• The data is designed for joint estimation of revealed and stated preferences.
• The data can be used to decompose willingness to pay into use and nonuse values.
• The data can be used to explore bid design and other differences in single and double-bounded willingness to pay.

## 1 Experimental design, materials and methods

The data are from a 1995 telephone survey conducted by the East Carolina University Survey Research Laboratory. The survey used a random digit dialing sampling scheme. The sample was purchased from Survey Sampling, Inc. and interviews were computer assisted. Of the households that were contacted, 1077 respondents provided data for an overall response rate of 75% [10].

There are two main versions of the telephone survey. Version 1 contained a contingent valuation method scenario for the Pamlico Sound and Version 2 contained a contingent valuation method scenario for the Albemarle and Pamlico Sounds. The main difference in the two versions is the insertion of “Albemarle and” before Pamlico in all questions and the addition of the plural to Sound(s). There are four types of questions in the contingent valuation method scenario.

The first group of questions elicited information about respondents׳ knowledge about and recreation participation and intensity on the Sound(s). The Pamlico Sound version began with: “Now I would like you to think about the Pamlico Sound, which is one of the large bodies of water in Eastern North Carolina near the Outer Banks. The Pungo, Tar-Pamlico, and Neuse Rivers flow into the Pamlico Sound. In general, how much do you know about the resources, uses, and problems of the Pamlico Sound? Would you say a lot (7% for both versions), some (15%), a little (32%), or nothing (42%)?” The Albemarle–Pamlico version differed only in that respondents were asked to think about the “… Albemarle and Pamlico Sounds, which are the large bodies of water in Eastern North Carolina near the Outer Banks. The Roanoke, Chowan, Perquimans, Pasquotank, and Alligator Rivers flow into the Albemarle Sound and the Pungo, Tar-Pamlico, and Neuse Rivers flow into the Pamlico Sound”.

Revealed preference and stated preference (i.e., contingent behavior) outdoor recreation participation was next elicited with the question: “Now I would like to ask you about any outdoor recreational activities you may have done on the Pamlico Sound. By recreational activities, I mean fishing, hunting, swimming, boating, skiing, windsurfing, birdwatching, camping, and so on. Did you participate in any recreational activities on or near the Pamlico Sound during the past 12 months?” Respondents who did participate (20%) were then asked “About how many trips did you take during the past 12 months?” and “About how many trips do you think you will take during the next 12 months?” Of those who participate, the average number of trips last year is 10 and 12 for next year. Respondents who did not participate were asked “Do you plan to participate in any recreational activities on or near the Pamlico Sound during the next 12 months?” and “About how many trips do you think you will take during the next 12 months?” Of those responding yes (*n*=49), the average number of trips is 3. Respondents in the Pamlico Sound version were asked about any other trips they took: “Other than at the Pamlico Sound, did you participate in any outdoor recreational activities during the past 12 months?” and “Where did you go for these trips?” Only 4% went to the Albemarle Sound while 59% went to the ocean/beach, 14% went to the mountains, 14% went to lakes, 13% went to rivers, and 18% went to other places. These numbers sum to greater than 100% due to multiple answers.

In the Albemarle–Pamlico version of the survey, if respondents had participated in recreation they were asked about current and future trips with the question “Where did you go for these trips, the Albemarle Sound (27%), the Pamlico Sound (41%), or both (32%)?” Respondents who did participate (20%) were then asked “About how many trips did you take during the past 12 months?” and “About how many trips do you think you will take during the next 12 months?” Of those who participate, the average number of trips last year is 13 and 17 for next year. All respondents were asked: “Other than at the Albemarle and Pamlico Sounds, did you participate in any outdoor recreational activities during the past 12 months?” Due to space limitations, only those respondents who had not participated in recreation on the Albemarle–Pamlico Sounds were asked where these trips took place (*n*=400), only 2% went to the Albemarle Sound, 64% went to the ocean/beach, 2% went to the mountains, 10% went to lakes, 9% went to rivers, and 14% went to other places. Respondents who did not participate were then asked “Do you plan to participate in any recreational activities on or near the Pamlico Sound during the next 12 months?” and “About how many trips do you think you will take during the next 12 months?” Of those responding yes (*n*=53), the average number of trips is 2.5.

The policy scenario was established next with a series of questions about respondents׳ concern about water pollution, support for tougher laws, and perceived effectiveness of these laws. These questions are designed to explain the pollution problem and the proposed policy and also to get respondents thinking about how much they value the policy. Again, for the Albemarle–Pamlico Version “Albemarle and Pamlico Sounds” was substituted for “Pamlico Sound”.

The pollution problem was described with the question: “Since 1981, fish catches have declined by over sixty percent and pollution has closed about twenty-five percent of the shellfish beds in the Pamlico Sound. How concerned are you about water pollution and damage to fish and wildlife habitat in the Pamlico Sound? Are you very concerned (46% for both versions), concerned (39%), slightly concerned (10%), or not concerned (5%)?”

The proposed water quality improvement policy was described as: “Chemicals, livestock waste, and soil erosion from farming can cause water pollution. Some commercial fishing practices, such as trawling and mechanical harvesting, can damage fish and wildlife habitat. Tougher laws that would require farmers to control pollution and that would restrict some fishing practices have been proposed for the Pamlico Sound. Do you strongly support (28% for both versions), support (62%), oppose (8%), or strongly oppose (2%) tougher pollution control laws?” The proposed change in water quality is a “restoration” of water quality and fish and wildlife habitat in the Sounds: “The goal of these laws would be to restore water quality and fish and wildlife habitat to the 1981 levels in the Pamlico Sound. How effective do you think these laws would be? Do you think they would be very effective (15% for both versions), somewhat effective (60%), slightly ineffective (20%), or not effective (6%)?”

The next question elicited information about stated preference recreation behavior with improved quality. Respondents were asked: “After enforcement of the tougher pollution control laws do you think that you would participate in any recreational activities on or near the (Albemarle and) Pamlico Sound(s) during the next 12 months?” If they answered “yes” (42% for Pamlico and 44% for A–P versions) they were then asked “About how many trips do you think you would take during the next 12 months?” The average number of trips was 6.6 and 8.5 for the Pamlico and A–P Versions. If they answered “no” they were asked why: “What is the main reason why you won׳t participate in any recreational activities?” with “not enough income” (2%), “too far to travel” (17%), “other places are better” (26%), “don׳t like recreation”(7%), “laws not effective” (*n*=1) and “water not clean enough”(1%) as possible answers while 47% gave some other reason.

The next series of questions established the contingent valuation scenario with double bounded dichotomous choice questions and reasons for the valuation responses. The payment obligation and payment vehicle were established with the following statement directly preceding the initial dichotomous choice question: “State government would need more tax money to enforce these tougher pollution control laws. Tougher pollution control laws would also mean higher consumer prices. It would cost you and your household about $PT1, each year, in higher prices and taxes. Remember, the goal would be to restore water quality and fish and wildlife habitat to 1981 levels in the Pamlico Sound only, other water bodies and wildlife habitat areas would not be affected. Would you be willing to pay $PT1, each year out of your own household budget, in higher prices and taxes?” The price and tax amounts, $PT1, were randomly selected from four amounts: 100, 200, 300, 400. If respondents answered “yes” (“no” or “don׳t know”) they were then asked the follow-up: “Would you be willing to pay $PT2 each year?” The value PT2 is equal to [2×PT1] if the first answer was “yes” and [.5×PT1] if “no”. Thirty-one percent of respondents answered “yes” to the initial question and 21% answered “yes” to the second question. Sixty percent of respondents answered “no” to the initial question and 67% answered “no” to the second question. Nine percent of respondents answered “don׳t know” to the initial question and 12% answered “don׳t know” to the second question.

If respondents answered either the initial or follow-up value elicitation question with a “yes,” the reasons for this value were probed with an open-ended question for which respondents could give as many reasons as they wanted. The initial answers given (*n*=344) were “for better recreation” (4%), “for future generations” (12%), “for friends and family” (2%), “for fish and wildlife” (29%), “it׳s the right thing to do (4%)”, “it sounds like a good cause (8%),” “I want a clean environment” (24%), or some other reason (8%). Respondents who gave as their initial answer reasons indicating that they were paying for moral satisfaction or warm glow (“it is the right thing to do”, “it sounds like a good cause”) were flagged as outliers. The answer “I want a clean environment” was also considered for flagging since it might indicate perceived payment for the environment in general and not for specific improvements in the A–P Sounds. These responses were not flagged after consideration of other reasons given for payment and respondent characteristics suggested that they did value specific improvements (i.e., they participate in recreation on the Pamlico Sound).

If the answers to both of the valuation questions were “no” the respondents then were asked: “What is the most important reason why you would not be willing to pay?” Answers given (*n*=642) include “cost is too high” (10%), “polluters should pay” (5%), “I don׳t trust government” (5%), “I׳m already paying enough in taxes (26%), “the environment is clean enough” (2%), “I don׳t like hypothetical questions” (*n*=1), “I don׳t have enough income” (10%), “I don׳t think the laws will be effective” (13%), “other areas are clean enough” (7%), or some other reason (15%). Most of the responses suggested that reasons related to high cost, budget constraints, or a lack of value for the improvement were the primary reasons for not being willing to pay. If respondents indicated that they rejected the payment vehicle or rule (“polluters should pay,” “I don׳t trust government”) or the contingent valuation scenario (“I don׳t like hypothetical questions,” I don׳t think the laws will be effective”) they were considered protest responses and flagged. The answer “I׳m already paying enough in taxes” was also considered for flagging since it might indicate rejection of the payment vehicle. An alternative interpretation is that these respondents could not afford to pay higher taxes so they were not flagged.

If the answers to both of the valuation questions were “don׳t know” the respondents then were asked: “What is the most important reason why you would not be willing to pay?” Answers given (*n*=78) include “cost is too high” (32%), “polluters should pay” (3%), “I don׳t trust government” (6%), “I׳m already paying enough in taxes" (15%), “I don׳t have enough income” (6%), “I don׳t think the laws will be effective” (*n*=1), “other areas are clean enough” (5%), or some other reason (19%). Protest responses were again flagged. Overall, 17% of respondents who answered at least “don׳t know” were flagged as protests or outliers for various reasons.

The demographic profile of the sample is similar to that of eastern North Carolina. The sample is 43% male, 54% married, 65% white, and 47% are employed full-time. The median age of the sample is 42 and the median education level is 13 years. Household income was elicited in categories. Twelve percent of households earned less than $10,000 and between $10,001 and $15,000, 11% of households earned between $15,001 and $20,000, 14% of households earned between $20,001 and $25,000, 10% earned between $25,001 and $30,000, 24% earned between $30,001 and $50,000, 12% earned between $50,001 and $75,000 and 5% earned above $75,001. With income levels coded at the midpoints of the income ranges (the upper range was coded as $85,000) the mean and median household income is $31,550 and $27,500.

For respondents living west of the Pamlico River, the recreation travel distance was calculated as the distance from the respondent׳s county population center to Washington, NC on the Pamlico River. If the respondent lived north or south of the Pamlico River, the distance was calculated as distance from the county population center to the nearest boat ramp on the Albemarle or Pamlico Sounds. Distances were calculated using the Automap software package.
