# Supplementary material for: Albemarle–Pamlico Sounds revealed and stated preference data
Source: Data Brief. 2015 Feb 13;3:90–4. doi: 10.1016/j.dib.2015.01.006 (PMC4510052; doi:10.1016/j.dib.2015.01.006)
Supplement: Supplementary file 1 — Supplementary Material [file mmc1.pdf]

1995 Albemarle-Pamlico Sounds Survey

Contingent Market Questions

There are two versions of the telephone survey:

Version 1: Pamlico Sound

Version 2: Albemarle and Pamlico Sounds

The questions are in six groups (for each version):

Q1	Knowledge. This question is designed to determine respondents knowledge about the Sound(s).
Q2, F1-F4, F5-F8	Recreation. These questions are designed to determine respondents recreation participation and intensity.
Q3-Q5	Contingent Market Set-Up. These questions are designed to explain the pollution problem and the proposed policy and also to get respondents thinking about their values for the policy.
Q6, F9, F10	Contingent Behavior. These questions are designed to get respondents thinking about how their behavior might change with the policy.
Q7, F11, F12	Contingent Valuation. These questions are designed to generate willingness to pay data with a follow-up response. There are four more versions (8 versions total).
F13, F14, F15	Debriefing. These questions are the standard follow ups to the contingent valuation questions.

Key:

Q=initial question
F=follow up

Version 1

Q1. Now I would like you to think about the Pamlico Sound, which is one of the large bodies of water in Eastern North Carolina near the Outer Banks. The Pungo, Tar-Pamlico, and Neuse Rivers flow into the Pamlico Sound. In general, how much do you know about the resources, uses, and problems of the Pamlico Sound? Would you say ...

A LOT
SOME
A LITTLE or
NOTHING

Version 2

Q1. Now I would like you to think about the **Albemarle and** Pamlico Sound**s**, which are the large bodies of water in Eastern North Carolina near the Outer Banks. **The Roanoke, Chowan, Perquimans, Pasquotank, and Alligator Rivers flow into the Albemarle Sound** and the Pungo, Tar-Pamlico, and Neuse Rivers flow into the Pamlico Sound. In general, how much do you know about the resources, uses, and problems of the Albemarle and Pamlico Sounds? Would you say ...

A LOT
SOME
A LITTLE
or NOTHING

Version 1

Q2. Now I would like to ask you about any outdoor recreational activities you may have done on the Pamlico Sound. By recreational activities, I mean fishing, hunting swimming, boating, skiing, windsurfing, birdwatching, camping, and so on. Did you participate in any recreational activities on or near the Pamlico Sound during the past 12 months?

YES [F1]
NO [F5]

<If recreation>

F1. About how many trips did you take during the past 12 months?

__________ TRIPS

F2. About how many trips do you think you will take during the next 12 months?

__________ TRIPS

F3. Other than at the Pamlico Sound, did you participate in any outdoor recreational activities during the past 12 months?

YES [F4]
NO [Q3]

F4. Where did you go for these trips? [DO NOT READ RESPONSES, CIRCLE ALL THAT ARE GIVEN]

ALBEMARLE SOUND
OCEAN/BEACH
MOUNTAINS
LAKES

RIVERS
OTHER

[Go to Q3.]

<If no recreation>

F5. Do you plan to participate in any recreational activities on or near the Pamlico Sound during the next 12 months?

YES [F6]
NO [F7]

F6. About how many trips do you think you will take during the next 12 months?

__________ TRIPS

F7. Other than at the Pamlico Sound, did you participate in any outdoor recreational activities during the past 12 months?

YES [F8]
NO [Q3]

F8. Where did you go for these trips? [DO NOT READ RESPONSES, CIRCLE ALL THAT ARE GIVEN]

ALBEMARLE SOUND
OCEAN/BEACH
MOUNTAINS
LAKES
RIVERS
OTHER

[Go to Q3.]

Version 2

Q2. Now I would like to ask you about any outdoor recreational activities you may have done on the Albemarle and Pamlico Sounds. By recreational activities, I mean fishing, hunting swimming, boating, skiing, windsurfing, birdwatching, camping, and so on. Did you participate in any recreational activities on or near the Albemarle or Pamlico Sounds during the past 12 months?

YES [F1]
NO [F5]

F1. About how many trips did you take during the past 12 months?

__________ TRIPS

F2. About how many trips do you think you will take during the next 12 months?

__________ TRIPS

F3. Where did you go for these trips? The Albemarle Sound, the Pamlico Sound, or both?

ALBEMARLE SOUND
PAMLICO SOUND
BOTH

F4. Other than at the Albemarle and Pamlico Sounds, did you participate in any outdoor recreational activities during the past 12 months?

YES
NO

F5. Do you plan to participate in any recreational activities on or near the Albemarle or Pamlico Sounds during the next 12 months?

YES [F6]
NO [F7]

F6. About how many trips do you think you will take during the next 12 months?

__________ TRIPS

F7. Other than at the Albemarle and Pamlico Sounds, did you participate in any outdoor recreational activities during the past 12 months?

YES [F8]
NO [Q3]

F8. Where did you go for these trips? [DO NOT READ RESPONSES, CIRCLE ALL THAT ARE GIVEN]

BAYS OR SOUNDS
OCEAN/BEACH
MOUNTAINS
LAKES
RIVERS
OTHER

[Go to Q3.]

Version 1

*<For version 2 substitute **Albemarle and Pamlico Sounds** for Pamlico Sound>*

Q3. Since 1981, fish catches have declined by over sixty percent and pollution has closed about twenty-five percent of the shellfish beds in the Pamlico Sound. How concerned are you about water pollution and damage to fish and wildlife habitat in the Pamlico Sound? Are you ...

VERY CONCERNED

CONCERNED
SLIGHTLY CONCERNED
or NOT CONCERNED

Q4. Chemicals, livestock waste, and soil erosion from farming can cause water pollution. Some commercial fishing practices, such as trawling and mechanical harvesting, can damage fish and wildlife habitat. Tougher laws that would require farmers to control pollution and that would restrict some fishing practices have been proposed for the Pamlico Sound. Do you ... tougher pollution control laws?

STRONGLY SUPPORT
SUPPORT
OPPOSE
STRONGLY OPPOSE

Q5. The goal of these laws would be to restore water quality and fish and wildlife habitat to the 1981 levels in the Pamlico Sound. How effective do you think these laws would be? Do you think they would be ...

VERY EFFECTIVE
EFFECTIVE
SLIGHTLY INEFFECTIVE
or NOT EFFECTIVE

Q6. After enforcement of the tougher pollution control laws do you think that you would participate in any recreational activities on or near the Pamlico Sound during the next 12 months?

YES [F9]
NO [F10]

F9. About how many trips do you think you would take during the next 12 months?

<if asked: we want to know the total number of trips during the year with improved water quality and wildlife habitat>

__________ TRIPS

F10. What is the main reason why you won't participate in any recreational activities? [DO NOT READ RESPONSES, CIRCLE ALL THAT ARE GIVEN]

NOT ENOUGH INCOME
OTHER PLACES ARE BETTER
TOO FAR TO TRAVEL
DON'T LIKE RECREATION
LAWS NOT EFFECTIVE
WATER NOT CLEAN ENOUGH
OTHER

Version 1

*<For version 2 substitute **Albemarle and Pamlico Sounds** for Pamlico Sound>*

Q7. State government would need more tax money to enforce these tougher pollution control laws. Tougher pollution control laws would also mean higher consumer prices. It would cost you and your household about $[PT], each year, in higher prices and taxes. Remember, the goal would be to restore water quality and fish and wildlife habitat to 1981 levels in the Pamlico Sound only, other water bodies and wildlife habitat areas would not be affected. Would you be willing to pay $[PT], each year out of your own household budget, in higher prices and taxes?

YES [F11]
NO [F12]
DON'T KNOW [F12]

F11. Would you be willing to pay $[2 x PT] each year?

YES [F13]
NO [F13]
DON'T KNOW [F13]

F12.Would you be willing to pay $[.5 x PT] each year?

YES [F13]
NO [F14]
DON'T KNOW [F14 or F15]

<[PT] is randomly selected from 100, 200, 300, 400>

<If answers to Q7 or F12 are YES>

F13. What is the most important reason why you would be willing to pay?

[DO NOT READ RESPONSES, CIRCLE ALL THAT ARE GIVEN]

FOR BETTER RECREATION
FOR FUTURE GENERATIONS
FOR FRIENDS AND FAMILY
FOR FISH AND WILDLIFE
IT IS THE RIGHT THING TO DO
I DON'T BELIEVE I'LL HAVE TO PAY
IT SOUNDS LIKE A GOOD CAUSE
I WANT A CLEAN ENVIRONMENT
or SOME OTHER REASON
DON'T KNOW

<If answers to Q7 and F12 are NO>

<If answers to Q7 is DK and F12 is NO>

F14. What is the most important reason why you would not be willing to pay?

[DO NOT READ RESPONSES, CIRCLE ALL THAT ARE GIVEN]

THE COST IS TOO HIGH
POLLUTERS SHOULD PAY
I DON'T TRUST GOVERNMENT
I'M ALREADY PAYING ENOUGH IN TAXES
THE ENVIRONMENT IS CLEAN ENOUGH
I DON'T LIKE HYPOTHETICAL QUESTIONS
I DON'T HAVE ENOUGH INCOME
I DON'T THINK THE LAWS WILL BE EFFECTIVE
OTHER AREAS ARE CLEAN ENOUGH
or SOME OTHER REASON
DON'T KNOW

<If answers to Q7 and F12 are DK>

F15. What is the most important reason why you do not know if you would be willing to pay [DO NOT READ RESPONSES, CIRCLE ALL THAT ARE GIVEN]

THE COST IS TOO HIGH
POLLUTERS SHOULD PAY
I DON'T TRUST GOVERNMENT
I'M ALREADY PAYING ENOUGH IN TAXES
THE ENVIRONMENT IS CLEAN ENOUGH
I DON'T LIKE HYPOTHETICAL QUESTIONS
I DON'T HAVE ENOUGH INCOME
I DON'T THINK THE LAWS WILL BE EFFECTIVE
OTHER AREAS ARE CLEAN ENOUGH
or SOME OTHER REASON
DON'T KNOW

<Go to demographic section of survey.>
